# Event-Level Association Between Daily Alcohol Use and Same-Day Nonadherence to Antiretroviral Therapy Among Young Men Who Have Sex With Men and Trans Women Living With HIV: Intensive Longitudinal Study

**DOI:** 10.2196/22733

**Published:** 2020-10-15

**Authors:** Caitlin Marie Turner, Dillon Trujillo, Victory Le, Erin C Wilson, Sean Arayasirikul

**Affiliations:** 1 Trans Research Unit for Equity Center for Public Health Research San Francisco Department of Public Health San Francisco, CA United States; 2 Doctoral Program in Epidemiology & Translational Science Department of Epidemiology and Biostatistics University of California San Francisco, CA United States; 3 California School of Professional Psychology Alliant International University San Francisco, CA United States; 4 Department of Epidemiology and Biostatistics University of California San Francisco, CA United States; 5 Department of Pediatrics School of Medicine University of California San Francisco, CA United States

**Keywords:** ecological momentary assessment, event-level, alcohol, antiretroviral therapy, men who have sex with men, trans women, adherence

## Abstract

**Background:**

Young trans women (TW) and men who have sex with men (MSM) are disproportionately impacted by HIV. Optimizing adherence to antiretroviral therapy (ART) is one mechanism by which public health experts aim to achieve favorable HIV health outcomes while reducing disease transmission. However, alcohol use is prevalent among young TW and MSM and threatens optimal adherence. In addition, the daily variations in alcohol use and ART adherence and their association with each other are poorly understood, warranting more appropriate methodological approaches, such as analysis of ecological momentary assessment (EMA) data.

**Objective:**

The aim of this analysis is to characterize the association between daily alcohol use and same-day ART nonadherence captured by an EMA study of young MSM and TW living with HIV in San Francisco.

**Methods:**

Young MSM and TW enrolled in the Health eNav digital HIV care navigation intervention were included in the analytic sample (N=113). Data on alcohol and ART use were collected by daily EMA surveys administered via text messaging and were analyzed over 30 days of follow-up. A multivariable mixed-effects logistic regression model adjusting for baseline sociodemographic characteristics was specified to investigate whether daily alcohol use was associated with same-day ART nonuse.

**Results:**

Daily alcohol use was associated with higher same-day ART nonuse. On average, participants drank alcohol on 15.20 (SD 8.93) days and used ART on 15.19 (SD 10.16) days out of 30 days. Daily alcohol use was associated with 1.89 (95% CI 1.14-3.15) times the adjusted odds of same-day ART nonuse for each participant.

**Conclusions:**

Results are consistent with other analyses of daily alcohol and ART use and underscore the importance of individually targeted interventions that are sensitive to each participant’s dynamic risk environment.

**International Registered Report Identifier (IRRID):**

RR2-10.2196/16406

## Introduction

Men who have sex with men (MSM) and trans women (TW) are disproportionately affected by HIV. MSM made up 70% of new HIV diagnoses in the United States in 2017 [1]. Approximately 21.7% of TW in the United States live with HIV and are over 30 times more likely to live with HIV compared to the rest of the US adult population [2].

The Joint United Nations Programme on HIV/AIDS (UNAIDS) developed 90-90-90 targets for 2020 to increase HIV care outcomes among all people living with HIV [3]. The last target—antiretroviral therapy (ART) uptake and adherence—is essential for viral suppression. Nonadherence to ART negatively impacts HIV progression [4,5] and is a substantial barrier to achieving favorable HIV health outcomes. Optimal ART adherence is achieved by only 63.4% of adults living with HIV [6,7].

With HIV-related stigma and other systems of oppression that target sexual and gender minority people, ART adherence is even poorer among MSM and TW. According to a 2018 HIV surveillance report by the Centers for Disease Control and Prevention (CDC), only 58% of gay and bisexual men living with HIV were virally suppressed [8]. In 2017, only 64.3% of TW in San Francisco had an undetectable viral load [9]. Low viral suppression among MSM and TW underscores the barriers to ART uptake and adherence for these populations.

Researchers have identified a number of intervention targets for improving ART adherence [5], including alcohol use [10,11]. Some mechanistic pathways involving alcohol use and ART adherence have been proposed. It is theorized that alcohol use impairs immunological functioning and viral suppression, and that this pathway is mediated by ART nonadherence [12,13]. Alcohol use could also impair cognitive functioning and subsequently interfere with the ability to adhere to HIV medications [14]. Alcohol use is prevalent among those living with HIV [15-18] and among MSM and TW [19-22], as it is hypothesized to be a coping strategy for psychological distress associated with HIV-related stigma [23] and discrimination based on sexual [24] or gender identity [25].

Alcohol use was associated with ART nonadherence in previous studies [10,11,26]; however, such studies are lacking among individuals living with HIV who have marginalized sexual and gender identities. Moreover, most studies of the effects of alcohol use on ART adherence are retrospective or gather data infrequently [27], failing to capture the temporality between alcohol and ART use and the day-to-day fluctuations inherent in both of these behaviors.

Ecological momentary assessment (EMA) data have the potential to characterize day-to-day or more frequent variations in health behaviors. EMA is a real-time data collection technique administered via technological platforms, such as handheld devices or mobile phones. A number of previous studies have used EMAs to capture fine-grained variations in substance use [28-31] and, more recently, behaviors among people living with HIV [28,32-34]. A number of studies have shown that EMA is feasible and acceptable among MSM [34-37] and persons who use substances [28,29]; one study showed moderate compliance to EMA among young MSM and TW living with HIV [38].

To our knowledge, 2 EMA studies were conducted to evaluate the relationship between alcohol use and ART adherence [39,40]. Parsons and colleagues [39] found that alcohol use was associated with 9 times the odds of HIV medication nonadherence among 272 HIV-positive men and women over 14 days of follow-up. Barai and colleagues [40] discovered that alcohol use was associated with lower odds of viral medication adherence and viral suppression among 234 women living with HIV. However, no studies have examined the alcohol use and ART nonadherence association in young MSM and TW living with HIV.

Given these research gaps, this study utilizes data from a relatively large sample of MSM and TW who participated in EMA as part of a larger HIV digital care navigation intervention. Using this data, we specify an intensive longitudinal model that assesses whether event-level alcohol use is associated with same-day ART nonadherence among young MSM and TW living with HIV in San Francisco.

## Methods

Data for this analysis come from the study of Health eNav, a Digital HIV care navigation intervention conducted at the San Francisco Department of Public Health from 2017 to 2018. Study procedures were approved by the University of California, San Francisco (IRB #16-19675). Health eNav is a digital HIV care navigation intervention that employs SMS text messaging to improve HIV care continuum outcomes for young MSM and TW living with HIV in San Francisco.

### Participants

Eligibility criteria were defined as follows: (1) self-identifying as MSM or TW; (2) aged 18-34 years; (3) living in San Francisco; and (4) newly diagnosed with HIV, not engaged/retained in care, or not virally suppressed. Specifically, we defined new HIV diagnoses as those that occurred within the last 12 months of enrollment in the study. If potential participants missed more than 2 HIV care appointments in the last year, they were considered as not engaged or retained in care. Potential participants who had a detectable viral load were considered as not virally suppressed. Potential participants were recruited via convenience sampling from 5 clinics and community-based organizations serving young people living with HIV in San Francisco. Study recruitment was advertised with posters, palm cards, and presentations; staff referred potential participants to the study through phone, email, or in-person meetings. The enrolled participants were also invited to refer peers from within their social networks.

An in-person or telephone eligibility screening was administered to recruits. Eligible participants then met with research staff situated at the local health department to obtain informed consent and enroll into the study. Overall, 171 people were screened. Out of those, 140 were eligible, and 120 participants enrolled in the study. Out of these 120 participants, 113 (94.2%) participants engaged in the EMA component of the study and comprised the analytical sample of this paper.

### Data Collection, Measures, and Variable Selection

Procedures for the Health eNav study are described in depth in a prior protocol [41]. Briefly, Health eNav was a 6-month digital HIV care navigation intervention among young MSM and TW living with HIV, a disproportionate number of whom experienced gaps in HIV care and subsequent disparities in ART use and viral suppression. All Health eNav participants were connected to a digital HIV care navigator who facilitated linkage to, engagement in, and retention in HIV care via 2-way SMS text messaging. This study analyzes data collected from the following sources:

1. Daily EMA text surveys delivered during the first 90 days of the larger intervention, focused on capturing daily substance use, affect, sexual behaviors, and ART use in the 24 hours prior to receiving each survey.
2. Computer-administered self-interviewing (CASI) surveys at baseline, 6-, 12-, and 18-month follow-up that gathered information on participants’ sociodemographic characteristics and HIV-related care outcomes.

We assessed the possible association between daily alcohol use and same-day ART adherence (measured by the EMA surveys) after adjusting for covariates (measured by baseline CASIs). We restricted our analysis to the first 30 days of EMA participation to be consistent with other EMA studies. Moreover, we observed that restricting to 30 days allowed us to maximize the nuance with which we characterized alcohol use and ART while minimizing any missingness due to participant fatigue.

Participants received automated SMS text message surveys once per day at the time of their choice (from among 8 AM, noon, or 8 PM) for the first 90 days of the intervention. The text survey was delivered through mSurvey [42]. Participants were required to complete EMA surveys within 24 hours, or the survey would time out. Each EMA survey comprised anywhere from 17 to 31 daily EMA texts depending on the responses. For example, if a participant reported on the EMA survey that they had sex within the last 24 hours, they would then receive follow-up questions about whether condoms were used and whether certain substances were used during sex. Had they not reported having sex, they would not receive questions about condom use or concurrent substance use. EMA surveys took 5 minutes or less to complete each day.

Participants were compensated US $1 for each completed EMA survey for up to US $90 over the EMA portion of the study. If participants completed more than 80% of their EMA surveys, they earned a bonus of US $100. Incentives were provided in the form of a gift card.

Data on alcohol use and ART adherence were collected daily for 30 days, and participants were inquired about their alcohol and ART use within the 24 hours prior to each EMA. We dichotomized daily alcohol use as “any use” versus “no use.” For ease with interpretation (since we hypothesized that alcohol use would be associated with nonuse of ART), we operationalized the outcome as daily ART nonuse compared to ART use (reference group). ART use was gathered from a question on the EMA survey that asked, “In the past 24 hours, did you take your ART meds?”

Factors that we hypothesized would confound the relationship between alcohol and ART use were selected a priori based on the creation of a directed acyclic graph. Age (in years); race/ethnicity (Black/African American, Hispanic/Latinx, other/multiple, or White); and education level (less than high school, high school or General Educational Development [GED], and at least some college) were included for adjustment in the main analytic model. We also included housing status (living with a family member, friend, or partner who rents/owns a home; living in temporary/transitional housing; experiencing homelessness; or renting/owning a home), recent incarceration, and competing needs (eg, foregoing HIV medications to afford basic needs such as food, housing, or clothing, and vice versa) as baseline covariates. We hypothesized that young TW and MSM who were recently diagnosed with HIV might experience heightened alcohol use and barriers to ART due to newly experienced stigma and identity development related to seroconversion. Therefore, we included HIV diagnosis timing (within the last 12 months of the baseline survey versus more than 12 months before baseline) as a covariate. Finally, we included number of substances (excluding alcohol or tobacco and including any combination of marijuana, heroin, methamphetamine, amphetamines, hallucinogens, crack/cocaine, heroin, opiates, or poppers) used at baseline, defined as 0, 1, or more than 1.

### Statistical Analysis

All analyses were conducted using Stata 14 software (StataCorp). Baseline sociodemographic characteristics, HIV diagnosis timing, and substance use, as gathered by the CASIs, were described for the entire sample. Alcohol use and ART nonuse were characterized over the 30-day period using EMA data. The main analysis, testing the association between daily alcohol use and same-day ART nonuse, comprised a mixed-effects regression model with a random intercept for each participant, adjusting for the aforementioned covariates.

## Results

Table 1 shows the demographic breakdown of participants in the analytic sample. The average age was about 28 years. Participants were racially/ethnically diverse, with most identifying as Black/African American, Hispanic/Latinx, or “Other”/multiple races, and about a quarter of the sample identifying as White. Though most participants had some college education or more (66/113, 58.4%), only a third of the sample rented or owned their living space. More than a quarter of the sample experienced competing needs (foregoing HIV medications to afford basic needs and vice versa). About a third of participants had been recently diagnosed with HIV, and the majority of participants reported using more than 2 substances (other than alcohol and tobacco) in the last 6 months.

Out of the 3390 total EMA surveys sent, 2022 (59.7%) were completed across Health eNav participants over the 30-day follow-up period. The median number of surveys completed, out of a total of 30 possible surveys, was 20 (IQR 8-27). There was a 97.85% overlap in missing values for ART and alcohol use; that is, most participants who did not respond to whether or not they used ART on a given day also did not respond to whether or not they used alcohol on that day. Alcohol use was reported on 19.1% of completed EMA surveys (386/2022); ART nonadherence was reported on 15.8% (320/2022) of completed EMA surveys.

**Table 1:** Baseline sociodemographic characteristics, HIV diagnosis timing, and substance use among young men who have sex with men and trans women living with HIV who participated in ecological momentary assessment text surveys over 30 days of follow-up, Health eNav (N=113), San Francisco, 2017-2019.

Sociodemographic characteristics			Values^a^
Age (years), mean (SD)			27.7 (3.96)
**Race/ethnicity, n (%)**			
	Black, non-Hispanic/Latinx	22 (19.47)	
	Hispanic/Latinx	37 (32.74)	
	Other or multiple races, non-Hispanic/Latinx	26 (23.01)	
	White, non-Hispanic/Latinx	28 (24.78)	
**Education level, n (%)**			
	Bachelor's or higher	11 (9.73)	
	Some college or Associate's degree	55 (48.67)	
	High school/GED^b^	36 (31.86)	
	Less than high school	11 (9.73)	
**Current living situation, n (%)**			
	Rent/own	36 (31.86)	
	Lives with a friend, partner, or family member	20 (17.70)	
	Temporary or transitional housing	41 (36.28)	
	Homeless/shelter	16 (14.16)	
**Went without HIV medications to afford basic needs, last 6 months, n (%)**			
	No	76 (67.26)	
	Yes	37 (32.74)	
**Went without basic needs to afford HIV medications, last 6 months, n (%)**			
	No	83 (73.45)	
	Yes	30 (26.55)	
**Incarcerated, last 6 months, n (%)**			
	No	94 (83.19)	
	Yes	19 (16.81)	
**HIV diagnosis timing (when diagnosed with HIV), n (%)**			
	Diagnosed more than 12 months prior to baseline	78 (69.03)	
	Diagnosed within 12 months prior to baseline survey	35 (30.97)	
**Substance use (number of substances used [other than alcohol and tobacco]) in last 6 months, n (%)**			
	0	22 (19.47)	
	1	24 (21.24)	
	2 or more	67 (59.29)	

^a^Percentages calculated out of the total number of participants who participated in EMA surveys (N=113), unless otherwise specified.

^b^GED: General Educational Development.

Analysis of EMA data revealed that, on average, participants drank alcohol on 15.20 (SD 8.93) days and used ART on 15.19 (SD 10.16) days out of 30 days. Event-level alcohol use was associated with 1.89 (95% CI 1.14-3.15) times the adjusted odds of same-day ART nonuse for each participant (Table 2).

**Table 2:** Mixed-effects model assessing daily alcohol use and same-day ART nonadherence among young MSM and TW living with HIV who participated in EMA text surveys over 30 days of follow-up, Health eNav (N=113), San Francisco, 2017-2019.

Variables			Mixed-effects logistic regression		
		AOR^a^ (95% CI)		*P* value	
**Exposure (used alcohol in the last 24 hours)**					
	No	Ref^b^		N/A^c^	
	Yes	1.89 (1.14-3.15)		.01	
Age			1.04 (0.92-1.19)		.52
**Race/ethnicity**					
	White, non-Hispanic/Latinx	Ref		N/A	
	Black, non-Hispanic/Latinx	3.97 (0.91-17.41)		.07	
	Hispanic/Latinx	0.94 (0.28-3.20)		.92	
	Other or multiple races, non-Hispanic/Latinx	0.27 (0.07-1.09)		.07	
**Education level**					
	Bachelor's or higher	Ref		N/A	
	Some college or Associate's degree	1.77 (0.35-9.10)		.49	
	High school/GED^d^	8.96 (1.54-52.15)		.02	
	Less than high school	2.25 (0.21-23.80)		.50	
**Current living situation**					
	Rent/own	Ref		N/A	
	Lives with a friend, partner, or family member	0.34 (0.08-1.44)		.14	
	Temporary or transitional housing	0.33 (0.10-1.11)		.07	
	Homeless/shelter	0.73 (0.16-3.37)		.68	
**Went without HIV medications to afford basic needs, last 6 months**					
	No	Ref		N/A	
	Yes	3.67 (1.06-12.71)		.04	
**Went without basic needs to afford HIV medications, last 6 months**					
	No	Ref		N/A	
	Yes	0.26 (0.07-1.04)		.06	
**Incarcerated, last 6 months**					
	No	Ref		N/A	
	Yes	0.77 (0.19-3.14)		.71	
**HIV diagnosis timing (when diagnosed with HIV)**					
	Diagnosed more than 12 months prior to baseline	Ref		N/A	
	Diagnosed within 12 months prior to baseline survey	0.28 (0.09-0.89)		.03	
**Substance use (number of substances used [other than alcohol and tobacco]) in last 6 months**					
	0	Ref		N/A	
	1	0.55 (0.12-2.51)		.44	
	2 or more	1.38 (0.40-4.71)		.61	

^a^AOR: adjusted odd ratios of same-day ART nonuse for daily alcohol use compared to nonuse, adjusting for age, race/ethnicity, education level, current living situation, competing needs, incarceration, HIV diagnosis timing, and substance use.

^b^Ref: reference.

^c^N/A: not applicable.

^d^GED: General Educational Development.

## Discussion

In summary, daily alcohol use was associated with higher same-day ART nonuse. Findings from this analysis corroborate other studies of the association between alcohol use and ART nonadherence, many of which collected data cross-sectionally or retrospectively. A meta-analysis of studies examining this association showed that the combined odds of ART adherence were lower among study participants who used alcohol. However, the authors noted that effect estimation would be improved with prospective, event-level examinations of alcohol and ART use and by assessing this association for different sociodemographic subgroups [43]. This analysis uses prospective, event-level data and examines this association for young MSM and TW living with HIV, 2 populations disproportionately affected by HIV. Showing a longitudinal association provides stronger evidence that interventions targeting alcohol use may also improve ART adherence, which is an important step in achieving optimal HIV care outcomes [3].

Findings from this study should be interpreted with a number of limitations in mind. First, intermittent missingness and dropout are issues inherent with EMA data collection due to increased burden on participants [44], and these issues were present in our sample. Full or partial multiple imputations have been recommended as the best approach for improving precision of estimates where missingness is an issue [44]. However, practical applications of these methods, especially with respect to longitudinal modeling, have produced mixed results. One study found that multiple imputations produced similar or even increased standard errors [45] compared with complete case data. With these observations in mind, we chose to analyze complete case data. Thus, our results are only generalizable to the participants for whom we had complete data.

Second, data were collected via self-report, and participants may have underreported their alcohol use and ART nonadherence behaviors. This could have potentially washed out the estimated effect. However, the results we observed still showed statistical significance. A third limitation was misclassification of exposure. Dichotomizing alcohol use as “any versus none” effectively grouped together participants who drank casually with those who drank heavily. However, such dichotomizing was necessary to preserve precision of estimates. In addition, we suspected that this misclassification was nondifferential and independent, which would bias results toward the null. If anything, the effect estimate we observed underestimated the true, underlying association. A fourth limitation was the small size of the sample, precluding stratified analyses by key demographics within which the alcohol-ART association may have varied. However, given the daily administration of EMA surveys, we are confident that the effect estimate provided was statistically precise for the entire sample. A fifth limitation was that we restricted the follow-up window of EMA surveys to 30 days. However, this analytic decision was informed by prior EMA studies conducted over 30-day-or-shorter time windows. Since EMA data collection was embedded within the larger digital care navigation intervention, losses-to-follow-up attributable to participation within the larger intervention may have impacted engagement in EMA outside of the 30-day window. Moreover, expanding the EMA follow-up window beyond 30 days could have produced an effect estimate that was less representative of the entire sample since participant fatigue and subsequent nonresponses were more of an issue beyond the first 30 days. Finally, results were not generalizable outside of young MSM and TW living in San Francisco.

The limitations of this analysis pave the way for future research. Given the dearth of research on moderation of the alcohol-ART association by key sociodemographic characteristics such as gender and race [43], future studies should utilize EMA methods in other key subgroups. TW living with HIV, many of whom experience pervasive gaps in HIV care and clinical outcomes, represented only a small percentage of the participants in this study, precluding statistically precise estimations of the effect of alcohol use on ART nonadherence for this subgroup. Future research should be conducted on larger samples of TW to explore how the alcohol-ART association varies by gender. Since this analysis was underpowered to assess interactions between alcohol use, substance use, and mental health, future studies with larger sample sizes could centralize comorbidities between those factors. In addition to focusing on individual behaviors, these future studies could examine the multilevel interplay between alcohol use and structural factors like racism, housing instability, or competing needs and their impact on ART adherence. Alcohol use is one of many modifiable factors that could affect adherence to HIV medications.

Finally, future EMA studies would benefit from a thorough consideration of institutional and individual barriers to EMA survey completion in order to reduce missing responses. A previous analysis of Health eNav EMA data confirmed that housing instability, incarceration, competing needs, and educational constraints interfered with EMA completion for young MSM and TW, even though participants had continuous access to cellular devices [38]. While one recommendation to preemptively address participant nonresponse would be to invest in procedures that sustain retention over the follow-up period, structural barriers to such an investment (eg, systemic marginalization of the populations served or lack of grant funding) highlight the unrealistic nature of such a recommendation. Dismantling systemic oppression would best improve study retention and even remove the need to have studies on health inequities in the first place. Until then, studies such as this one highlight the need to apply tailored approaches to implementation of digital interventions within under- and misrepresented populations.

To our knowledge, this is the first intensive longitudinal analysis of alcohol and ART use among young MSM and TW living with HIV. This analysis highlights important considerations in examining daily ART use among populations especially vulnerable to substance use and medication nonadherence.

## Abbreviations

ART: antiretroviral therapy
CASI: computer-administered self-interviewing
CDC: Centers for Disease Control and Prevention
EMA: ecological momentary assessment
GED: General Educational Development
MSM: men who have sex with men
TW: trans women
UNAIDS: Joint United Nations Programme on HIV/AIDS
